# Big Data: Survey, Technologies, Opportunities, and Challenges

**DOI:** 10.1155/2014/712826

**Published:** 2014-07-17

**Authors:** Nawsher Khan, Ibrar Yaqoob, Ibrahim Abaker Targio Hashem, Zakira Inayat, Waleed Kamaleldin Mahmoud Ali, Muhammad Alam, Muhammad Shiraz, Abdullah Gani

**Affiliations:** ^1^Mobile Cloud Computing Research Lab, Faculty of Computer Science and Information Technology, University of Malaya, 50603 Kuala Lumpur, Malaysia; ^2^Department of Computer Science, Abdul Wali Khan University Mardan, Mardan 23200, Pakistan; ^3^Department of Computer Science, University of Engineering and Technology Peshawar, Peshawar 2500, Pakistan; ^4^Saudi Electronic University, Riyadh, Saudi Arabia; ^5^Universiti Kuala Lumpur, 50603 Kuala Lumpur, Malaysia

## Abstract

Big Data has gained much attention from the academia and the IT industry. In the digital and computing world, information is generated and collected at a rate that rapidly exceeds the boundary range. Currently, over 2 billion people worldwide are connected to the Internet, and over 5 billion individuals own mobile phones. By 2020, 50 billion devices are expected to be connected to the Internet. At this point, predicted data production will be 44 times greater than that in 2009. As information is transferred and shared at light speed on optic fiber and wireless networks, the volume of data and the speed of market growth increase. However, the fast growth rate of such large data generates numerous challenges, such as the rapid growth of data, transfer speed, diverse data, and security. Nonetheless, Big Data is still in its infancy stage, and the domain has not been reviewed in general. Hence, this study comprehensively surveys and classifies the various attributes of Big Data, including its nature, definitions, rapid growth rate, volume, management, analysis, and security. This study also proposes a data life cycle that uses the technologies and terminologies of Big Data. Future research directions in this field are determined based on opportunities and several open issues in Big Data domination. These research directions facilitate the exploration of the domain and the development of optimal techniques to address Big Data.

## 1. Introduction

The current international population exceeds 7.2 billion [1], and over 2 billion of these people are connected to the Internet. Furthermore, 5 billion individuals are using various mobile devices, according to McKinsey (2013). As a result of this technological revolution, these millions of people are generating tremendous amounts of data through the increased use of such devices. In particular, remote sensors continuously produce much heterogeneous data that are either structured or unstructured. This data is known as Big Data [2]. Big Data is characterized by three aspects: (a) the data are numerous, (b) the data cannot be categorized into regular relational databases, and (c) data are generated, captured, and processed very quickly. Big Data is promising for business application and is rapidly increasing as a segment of the IT industry. It has generated significant interest in various fields, including the manufacture of healthcare machines, banking transactions, social media, and satellite imaging [3]. Traditionally, data is stored in a highly structured format to maximize its informational contents. However, current data volumes are driven by both unstructured and semistructured data. Therefore, end-to-end processing can be impeded by the translation between structured data in relational systems of database management and unstructured data for analytics.

e staggering growth rate of the amount of collected data generates numerous critical issues and challenges described by [4], such as rapid data growth, transfer speed, diverse data, and security issues. Nonetheless, the advancements in data storage and mining technologies enable the preservation of these increased amounts of data. In this preservation process, the nature of the data generated by organizations is modified [5]. However, Big Data is still in its infancy stage and has not been reviewed in general. Hence, this study comprehensively surveys and classifies the various attributes of Big Data, including its volume, management, analysis, security, nature, definitions, and rapid growth rate. The study also proposes a data life cycle that uses the technologies and terminologies of Big Data. Future research directions in this field are determined by opportunities and several open issues in Big Data domination.

This study presents: (a) a comprehensive survey of Big Data characteristics; (b) a discussion of the tools of analysis and management related to Big Data; (c) the development of a new data life cycle with Big Data aspects; and (d) an enumeration of the issues and challenges 5 associated with Big Data.

The rest of the paper is organized as follows.  Section 2 explains fundamental concepts and describes the rapid growth of data volume; Section 3 discusses the management of Big Data and the related tools; Section 4 proposes a new data life cycle that utilizes the technologies and terminologies of Big Data; Section 5 describes the opportunities, open issues, and challenges in this domain; and Section 6 concludes the paper. Lists of acronyms used in this paper are presented in the Acronyms section.

## 2. Background

Information increases rapidly at a rate of 10x every five years [6]. From 1986 to 2007, the international capacities for technological data storage, computation, processing, and communication were tracked through 60 analogues and digital technologies [7, 8]; in 2007, the capacity for storage in general-purpose computers was 2.9 × 10^20^ bytes (optimally compressed) and that for communication was 2.0 × 10^21^ bytes. These computers could also accommodate 6.4 × 10^18^ instructions per second [7]. However, the computing size of general-purpose computers increases annually at a rate of 58% [7]. In computational sciences, Big Data is a critical issue that requires serious attention [9, 10]. Thus far, the essential landscapes of Big Data have not been unified. Furthermore, Big Data cannot be processed using existing technologies and methods [7]. Therefore, the generation of incalculable data by the fields of science, business, and society is a global problem. With respect to data analytics, for instance, procedures and standard tools have not been designed to search and analyze large datasets [8]. As a result, organizations encounter early challenges in creating, managing, and manipulating large datasets. Systems of data replication have also displayed some security weaknesses with respect to the generation of multiple copies, data governance, and policy. These policies define the data that are stored, analyzed, and accessed. They also determine the relevance of these data. To process unstructured data sources in Big Data projects, concerns regarding the scalability, low latency, and performance of data infrastructures and their data centers must be addressed [11].

**Figure 1:**
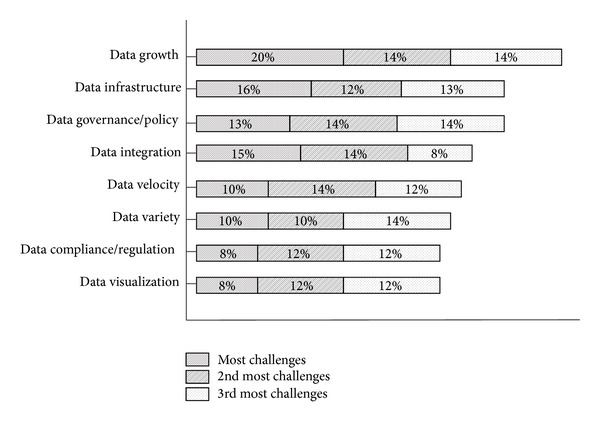
Challenges in Big Data [13].

In the IT industry as a whole, the rapid rise of Big Data has generated new issues and challenges with respect to data management and analysis. Five common issues are volume, variety, velocity, value, and complexity according to [4, 12]. In this study, there are additional issues related to data, such as the fast growth of volume, variety, value, management, and security. Each issue represents a serious problem of technical research that requires discussion. Hence, this research proposes a data life cycle that uses the technologies and terminologies of Big Data. Future research directions in this field are determined based on opportunities and several open issues in Big Data domination.  Figure 1 [13] groups the critical issues in Big Data into three categories based on the commonality of the challenge.

### 2.1. Volume of Big Data

The volume of Big Data is typically large. However, it does not require a certain amount of petabytes. The increase in the volume of various data records is typically managed by purchasing additional online storage; however, the relative value of each data point decreases in proportion to aspects such as age, type, quantity, and richness. Thus, such expenditure is unreasonable (Doug, 212). The following two subsections detail the volume of Big Data in relation to the rapid growth of data and the development rate of hard disk drives (HDDs). It also examines Big Data in the current environment of enterprises and technologies.

#### 2.1.1. Rapid Growth of Data

The data type that increases most rapidly is unstructured data. This data type is characterized by “human information” such as high-definition videos, movies, photos, scientific simulations, financial transactions, phone records, genomic datasets, seismic images, geospatial maps, e-mail, tweets, Facebook data, call-center conversations, mobile phone calls, website clicks, documents, sensor data, telemetry, medical records and images, climatology and weather records, log files, and text [11]. According to Computer World, unstructured information may account for more than 70% to 80% of all data in organizations [14]. These data, which mostly originate from social media, constitute 80% of the data worldwide and account for 90% of Big Data. Currently, 84% of IT managers process unstructured data, and this percentage is expected to drop by 44% in the near future [11]. Most unstructured data are not modeled, are random, and are difficult to analyze. For many organizations, appropriate strategies must be developed to manage such data.  Table 1 describes the rapid production of data in various organizations further.

According to Industrial Development Corporation (IDC) and EMC Corporation, the amount of data generated in 2020 will be 44 times greater [40 zettabytes (ZB)] than in 2009. This rate of increase is expected to persist at 50% to 60% annually [21]. To store the increased amount of data, HDDs must have large storage capacities. Therefore, the following section investigates the development rate of HDDs.

#### 2.1.2. Development Rate of Hard Disk Drives (HDDs)

**Figure 2:**
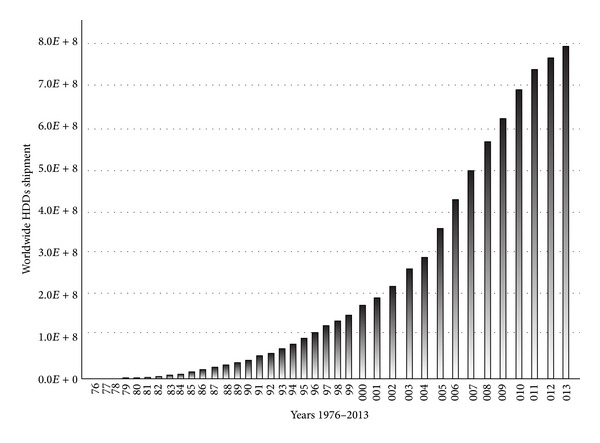
Worldwide shipment of HDDs from 1976 to 2013.

The demand for digital storage is highly elastic. It cannot be completely met and is controlled only by budgets and management capability and capacity. Goda et al. (2002) and [22] discuss the history of storage devices, starting with magnetic tapes and disks and optical, solid-state, and electromechanical devices. Prior to the digital revolution, information was predominantly stored in analogue videotapes according to the available bits. As of 2007, however, most data are stored in HDDs (52%), followed by optical storage (28%) and digital tapes (roughly 11%). Paper-based storage has dwindled 0.33% in 1986 to 0.007% in 2007, although its capacity has steadily increased (from 8.7 optimally compressed PB to 19.4 optimally compressed PB) [22].  Figure 2 depicts the rapid development of HDDs worldwide.

The HDD is the main component in electromechanical devices. In 2013, the expected revenue from global HDDs shipments was $33 billion, which was down 12% from the predicted $37.8 billion in 2012 [23]. Furthermore, data regarding the quantity of units shipped between 1976 and 1998 was obtained from Datasheetcatalog.com, 1995 [24]; [25–27]; Mandelli and Bossi, 2002 [28]; MoHPC, 2003; Helsingin Sanomat, 2000 [29]; Belk, 2007 [30–33]; and J. Woerner, 2010; those shipped between 1999 and 2004 were provided by Freescale Semiconductors 2005 [34, 35]; PortalPlayer, 2005 [36]; NVIDIA, 2009 [37, 38]; and Jeff, 1997 [39]; those shipped in 2005 and 2006 were obtained from Securities and Exchange Commission, 1998 [40]; those shipped in 2007 were provided by [41–43]; and those shipped from 2009 to 2013 were obtained from [23]. Based on the information gathered above, the quantity of HDDs shipped will exceed 1 billion annually by 2016 given a progression rate of 14% from 2014 to 2016 [23]. As presented in Figure 2, the quantities of HDDs shipped per year were 175.7*E* + 3, 493.5*E* + 3, 27879.1*E* + 3, 195451*E* + 3, and 779579*E* + 3 in 1976, 1980, 1990, 2000, and 2012, respectively. According to Coughlin Associates, HDDs expenditures are expected to increase by 169% from 2011 to 2016, thus affecting the current enterprise environment significantly. Given this finding, the following section discusses the role of Big Data in the current enterprise environment.

### 2.2. Big Data in the Current Environments of Enterprise and Technology

In 2012, 2.5 quintillion bytes of data were generated daily, and 90% of current data worldwide originated in the past two years ([20] and Big Data, 2013). During 2012, 2.2 million TB of new data are generated each day. In 2010, the market for Big Data was $3.2 billion, and this value is expected to increase to $16.9 billion in 2015 [20]. As of July 9, 2012, the amount of digital data in the world was 2.7 ZB [11]; Facebook alone stores, accesses, and analyzes 30 + PB of user-generated data [16]. In 2008, Google was processing 20,000 TB of data daily [44]. To enhance advertising, Akamai processes and analyzes 75 million events per day [45]. Walmart processes over 1 million customer transactions, thus generating data in excess of 2.5 PB as an estimate.

More than 5 billion people worldwide call, text, tweet, and browse on mobile devices [46]. The amount of e-mail accounts created worldwide is expected to increase from 3.3 billion in 2012 to over 4.3 billion by late 2016 at an average annual rate of 6% over the next four years. In 2012, a total of 89 billion e-mails were sent and received daily, and this value is expected to increase at an average annual rate of 13% over the next four years to exceed 143 billion by the end of 2016 [47]. In 2012, 730 million users (34% of all e-mail users) were e-mailing through mobile devices. Boston.com [47] reported that in 2013, approximately 507 billion e-mails were sent daily. Currently, an e-mail is sent every 3.5 × 10^−7^ seconds. Thus, the volume of data increases per second as a result of rapid data generation.

Growth rates can be observed based on the daily increase in data. Until the early 1990s, annual growth rate was constant at roughly 40%. After this period, however, the increase was sharp and peaked at 88% in 1998 [7]. Technological progress has since slowed down. In late 2011, 1.8 ZB of data were created as of that year, according to IDC [21]. In 2012, this value increased to 2.8 ZB. Globally, approximately 1.2 ZB (10^21^) of electronic data are generated per year by various sources [7]. By 2020, enterprise data is expected to total 40 ZB, as per IDC [12]. Based on this estimation, business-to-consumer (B2C) and internet-business-to-business (B2B) transactions will amount to 450 billion per day. Thus, efficient management tools and techniques are required.

## 3. Big Data Management

The architecture of Big Data must be synchronized with the support infrastructure of the organization. To date, all of the data used by organizations are stagnant. Data is increasingly sourced from various fields that are disorganized and messy, such as information from machines or sensors and large sources of public and private data. Previously, most companies were unable to either capture or store these data, and available tools could not manage the data in a reasonable amount of time. However, the new Big Data technology improves performance, facilitates innovation in the products and services of business models, and provides decision-making support [8, 48]. Big Data technology aims to minimize hardware and processing costs and to verify the value of Big Data before committing significant company resources. Properly managed Big Data are accessible, reliable, secure, and manageable. Hence, Big Data applications can be applied in various complex scientific disciplines (either single or interdisciplinary), including atmospheric science, astronomy, medicine, biology, genomics, and biogeochemistry. In the following section, we briefly discuss data management tools and propose a new data life cycle that uses the technologies and terminologies of Big Data.

### 3.1. Management Tools

With the evolution of computing technology, immense volumes can be managed without requiring supercomputers and high cost. Many tools and techniques are available for data management, including Google BigTable, Simple DB, Not Only SQL (NoSQL), Data Stream Management System (DSMS), MemcacheDB, and Voldemort [3]. However, companies must develop special tools and technologies that can store, access, and analyze large amounts of data in near-real time because Big Data differs from the traditional data and cannot be stored in a single machine. Furthermore, Big Data lacks the structure of traditional data. For Big Data, some of the most commonly used tools and techniques are Hadoop, MapReduce, and Big Table. These innovations have redefined data management because they effectively process large amounts of data efficiently, cost-effectively, and in a timely manner. The following section describes Hadoop and MapReduce in further detail, as well as the various projects/frameworks that are related to and suitable for the management and analysis of Big Data.

### 3.2. Hadoop

Hadoop [49] is written in Java and is a top-level Apache project that started in 2006. It emphasizes discovery from the perspective of scalability and analysis to realize near-impossible feats. Doug Cutting developed Hadoop as a collection of open-source projects on which the Google MapReduce programming environment could be applied in a distributed system. Presently, it is used on large amounts of data. With Hadoop, enterprises can harness data that was previously difficult to manage and analyze. Hadoop is used by approximately 63% of organizations to manage huge number of unstructured logs and events (Sys.con Media, 2011).

In particular, Hadoop can process extremely large volumes of data with varying structures (or no structure at all). The following section details various Hadoop projects and their links according to [12, 50–55].

**Figure 3:**
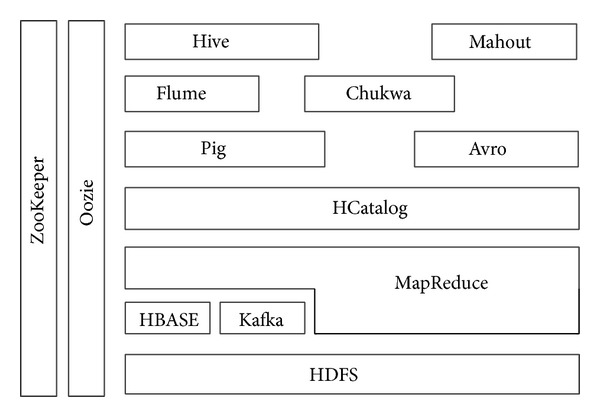
Hadoop ecosystem.

Hadoop is composed of HBase, HCatalog, Pig, Hive, Oozie, Zookeeper, and Kafka; however, the most common components and well-known paradigms are Hadoop Distributed File System (HDFS) and MapReduce for Big Data.  Figure 3 illustrates the Hadoop ecosystem, as well as the relation of various components to one another.

*HDFS.* This paradigm is applied when the amount of data is too much for a single machine. HDFS is more complex than other file systems given the complexities and uncertainties of networks. Cluster contains two types of nodes. The first node is a name-node that acts as a master node. The second node type is a data node that acts as slave node. This type of node comes in multiples. Aside from these two types of nodes, HDFS can also have secondary name-node. HDFS stores files in blocks, the default block size of which is 64 MB. All HDFS files are replicated in multiples to facilitate the parallel processing of large amounts of data. 

*HBase.* HBase is a management system that is open-source, versioned, and distributed based on the BigTable of Google. This system is column- rather than row-based, which accelerates the performance of operations over similar values across large data sets. For example, read and write operations involve all rows but only a small subset of all columns. HBase is accessible through application programming interfaces (APIs) such as Thrift, Java, and representational state transfer (REST). These APIs do not have their own query or scripting languages. By default, HBase depends completely on a ZooKeeper instance.

*ZooKeeper.* ZooKeeper maintains, configures, and names large amounts of data. It also provides distributed synchronization and group services. This instance enables distributed processes to manage and contribute to one another through a name space of data registers (*z*-nodes) that is shared and hierarchical, such as a file system. Alone, ZooKeeper is a distributed service that contains* master* and* slave* nodes and stores configuration information.

*HCatalog.* HCatalog manages HDFS. It stores metadata and generates tables for large amounts of data. HCatalog depends on Hive metastore and integrates it with other services, including MapReduce and Pig, using a common data model. With this data model, HCatalog can also expand to HBase. HCatalog simplifies user communication using HDFS data and is a source of data sharing between tools and execution platforms.

*Hive.* Hive structures warehouses in HDFS and other input sources, such as Amazon S3. Hive is a subplatform in the Hadoop ecosystem and produces its own query language (HiveQL). This language is compiled by MapReduce and enables user-defined functions (UDFs). The Hive platform is primarily based on three related data structures: tables, partitions, and buckets. Tables correspond to HDFS directories and can be distributed in various partitions and, eventually, buckets. 

*Pig.* The Pig framework generates a high-level scripting language (Pig Latin) and operates a run-time platform that enables users to execute MapReduce on Hadoop. Pig is more elastic than Hive with respect to potential data format given its data model. Pig has its own data type,* map*, which represents semistructured data, including JSON and XML. 

*Mahout.* Mahout is a library for machine-learning and data mining. It is divided into four main groups: collective filtering, categorization, clustering, and mining of parallel frequent patterns. The Mahout library belongs to the subset that can be executed in a distributed mode and can be executed by MapReduce. 

*Oozie.* In the Hadoop system, Oozie coordinates, executes, and manages job flow. It is incorporated into other Apache Hadoop frameworks, such as Hive, Pig, Java MapReduce, Streaming MapReduce, and Distcp Sqoop. Oozie combines actions and arranges Hadoop tasks using a directed acyclic graph (DAG). This model is commonly used for various tasks. 

*Avro.* Avro serializes data, conducts remote procedure calls, and passes data from one program or language to another. In this framework, data are self-describing and are always stored based on their own schema because these qualities are particularly suited to scripting languages such as Pig. 

*Chukwa.* Currently, Chukwa is a framework for data collection and analysis that is related to MapReduce and HDFS. This framework is currently progressing from its development stage. Chukwa collects and processes data from distributed systems and stores them in Hadoop. As an independent module, Chukwa is included in the distribution of Apache Hadoop. 

*Flume.* Flume is specially used to aggregate and transfer large amounts of data (i.e., log data) in and out of Hadoop. It utilizes two channels, namely,* sources* and* sinks*. Sources include Avro, files, and system logs, whereas sinks refer to HDFS and HBase. Through its personal engine for query processing, Flume transforms each new batch of Big Data before it is shuttled into the sink.

Table 2 summarizes the functionality of the various Hadoop components discussed above.

Hadoop is widely used in industrial applications with Big Data, including spam filtering, network searching, clickstream analysis, and social recommendation. To distribute its products and services, such as spam filtering and searching, Yahoo has run Hadoop in 42,000 servers at four data centers as of June 2012. Currently, the largest Hadoop cluster contains 4,000 nodes, which is expected to increase to 10,000 with the release of Hadoop 2.0 [3]. Simultaneously, Facebook announced that their Hadoop cluster processed 100 PB of data, which increased at a rate of 0.5 PB per day as of November 2012. According to Wiki, 2013, some well-known organizations and agencies also use Hadoop to support distributed computations (Wiki, 2013). In addition, various companies execute Hadoop commercially and/or provide support, including Cloudera, EMC, MapR, IBM, and Oracle.

With Hadoop, 94% of users can analyze large amounts of data. Eighty-eight percent of users analyze data in detail, and 82% can retain more data (Sys.con Media, 2011). Although Hadoop has various projects (Table 2), each company applies a specific Hadoop product according to its needs. Thus, Facebook stores 100 PB of both structured and unstructured data using Hadoop. IBM, however, primarily aims to generate a Hadoop platform that is highly accessible, scalable, effective, and user-friendly. It also seeks to flatten the time-to-value curve associated with Big Data analytics by establishing development and runtime environments for advanced analytical application and to provide Big Data analytic tools for business users.  Table 3 presents the specific usage of Hadoop by companies and their purposes.

**Figure 4:**
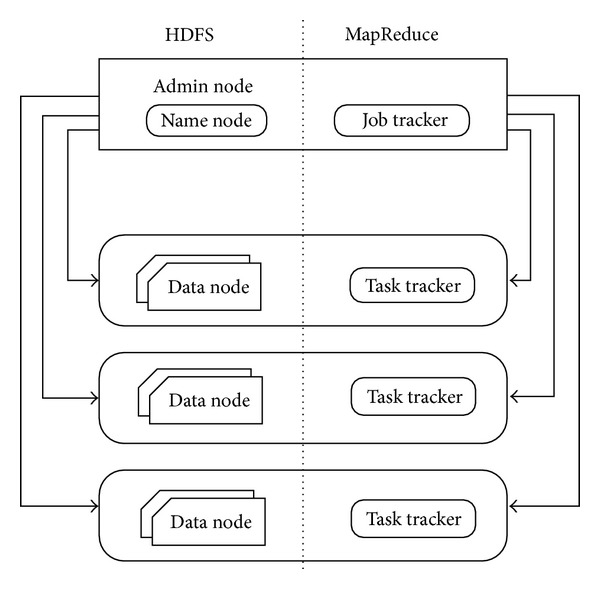
System architectures of MapReduce and HDFS.

To scale the processing of Big Data, map and reduce functions can be performed on small subsets of large datasets [56, 57]. In a Hadoop cluster, data are deconstructed into smaller blocks. These blocks are distributed throughout the cluster. HDFS enables this function, and its design is heavily inspired by the distributed file system Google File System (GFS).  Figure 4 depicts the architectures of MapReduce and HDFS.

MapReduce is the hub of Hadoop and is a programming paradigm that enables mass scalability across numerous servers in a Hadoop cluster. In this cluster, each server contains a set of internal disk drives that are inexpensive. To enhance performance, MapReduce assigns workloads to the servers in which the processed data are stored. Data processing is scheduled based on the cluster nodes. A node may be assigned a task that requires data foreign to that node. The functionality of MapReduce has been discussed in detail by [56, 57].

MapReduce actually corresponds to two distinct jobs performed by Hadoop programs. The first is the map job, which involves obtaining a dataset and transforming it into another dataset. In these datasets, individual components are deconstructed into tuples (key/value pairs). The reduction task receives inputs from map outputs and further divides the data tuples into small sets of tuples. Therefore, the reduction task is always performed after the map job.  Table 4 introduces MapReduce tasks in job processing step by step.

**Figure 5:**
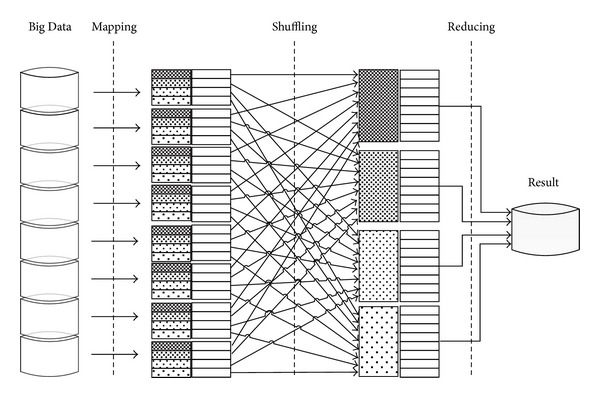
MapReduce architecture.

Redundant data are stored in multiple areas across the cluster. The programming model resolves failures automatically by running portions of the program on various servers in the cluster. Data can be distributed across a very large cluster of commodity components along with associated programming given the redundancy of data. This redundancy also tolerates faults and enables the Hadoop cluster to repair itself if the component of commodity hardware fails, especially given large amount of data. With this process, Hadoop can delegate workloads related to Big Data problems across large clusters of reasonable machines.  Figure 5 shows the MapReduce architecture.

### 3.3. Limitations of Hadoop

With Hadoop, extremely large volumes of data with either varying structures or none at all can be processed, managed, and analyzed. However, Hadoop also has some limitations. 

*The Generation of Multiple Copies of Big Data.* HDFS was built for efficiency; thus, data is replicated in multiples. Generally, data are generated in triplicate at minimum. However, six copies must be generated to sustain performance through data locality. As a result, the Big Data is enlarged further. 

*Challenging Framework.* The MapReduce framework is complicated, particularly when complex transformational logic must be leveraged. Attempts have been generated by open-source modules to simplify this framework, but these modules also use registered languages. 

*Very Limited SQL Support.* Hadoop combines open-source projects and programming frameworks across a distributed system. Consequently, offers it gains limited SQL support and lacks basic SQL functions, such as subqueries and grouping by analytics. 

*Lack of Essential Skills.* Intriguing data mining libraries are implemented inconsistently as part of the Hadoop project. Thus, algorithm knowledge and development skill with respect to distributed MapReduce are necessary. 

*Inefficient Execution.* HDFS does not consider query optimizers. Therefore, it cannot execute an efficient cost-based plan. Hence, the sizes of Hadoop clusters are often significantly larger than needed for a similar database.

## 4. Life Cycle and Management of Data Using Technologies and Terminologies of Big Data

During each stage of the data life cycle, the management of Big Data is the most demanding issue. This problem was first raised in the initiatives of UK e-Science a decade ago. In this case, data were geographically distributed, managed, and owned by multiple entities [4]. The new approach to data management and handling required in e-Science is reflected in the scientific data life cycle management (SDLM) model. In this model, existing practices are analyzed in different scientific communities. The generic life cycle of scientific data is composed of sequential stages, including experiment planning (research project), data collection and processing, discussion, feedback, and archiving [58–60].

**Figure 6:**
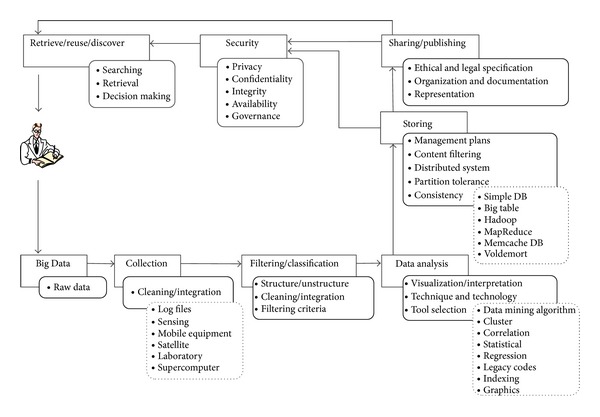
Proposed data life cycle using the technologies and terminologies of Big Data.

The following section presents a general data life cycle that uses the technology and terminology of Big Data. The proposed data life cycle consists of the following stages: collection, filtering & classification, data analysis, storing, sharing & publishing, and data retrieval & discovery. The following sections briefly describe each stage as exhibited in Figure 6.

### 4.1. Raw Data

Researchers, agencies, and organizations integrate the collected raw data and increase their value through input from individual program offices and scientific research projects. The data are transformed from their initial state and are stored in a value-added state, including web services. Neither a benchmark nor a globally accepted standard has been set with respect to storing raw data and minimizing data. The code generates the data along with selected parameters.

### 4.2. Collection/Filtering/Classification

Data collection or generation is generally the first stage of any data life cycle. Large amounts of data are created in the forms of log file data and data from sensors, mobile equipment, satellites, laboratories, supercomputers, searching entries, chat records, posts on Internet forums, and microblog messages. In data collection, special techniques are utilized to acquire raw data from a specific environment. A significant factor in the management of scientific data is the capture of data with respect to the transition of raw to published data processes. Data generation is closely associated with the daily lives of people. These data are also similarly of low density and high value. Normally, Internet data may not have value; however, users can exploit accumulated Big Data through useful information, including user habits and hobbies. Thus, behavior and emotions can be forecasted. The problem of scientific data is one that must be considered by Scientific Data Infrastructure (SDI) providers [58, 59] . In the following paragraphs, we explain five common methods of data collection, along with their technologies and techniques. 

*(i) Log Files.* This method is commonly used to collect data by automatically recording files through a data source system. Log files are utilized in nearly all digital equipment; that is, web servers note the number of visits, clicks, click rates, and other property records of web users in log files [61]. In web sites and servers, user activity is captured in three log file formats (all are in ASCII): (i) public log file format (NCSA); (ii) expanded log format (W3C); and (iii) IIS log format (Microsoft). To increase query efficiency in massive log stores, log information is occasionally stored in databases rather than text files [62, 63]. Other log files that collect data are stock indicators in financial applications and files that determine operating status in network monitoring and traffic management.

*(ii) Sensing*. Sensors are often used to measure physical quantities, which are then converted into understandable digital signals for processing and storage. Sensory data may be categorized as sound wave, vibration, voice, chemical, automobile, current, pressure, weather, and temperature. Sensed data or information is transferred to a collection point through wired or wireless networks. The wired sensor network obtains related information conveniently for easy deployment and is suitable for management applications, such as video surveillance system [64].

When position is inaccurate, when a specific phenomenon is unknown, and when power and communication have not been set up in the environment, wireless communication can enable data transmission within limited capabilities. Currently, the wireless sensor network (WSN) has gained significant attention and has been applied in many fields, including environmental research [65, 66], the monitoring of water quality [67], civil engineering [68, 69], and the tracking of wildlife habit [70]. The data through any application is assembled in various sensor nodes and sent back to the base location for further handling. Sensed data have been discussed by [71] in detail. 

*(iii) Methods of Network Data Capture.* Network data is captured by combining systems of web crawler, task, word segmentation, and index. In search engines, web crawler is a component that downloads and stores web pages [72]. It obtains access to other linked pages through the Uniform Resource Locator (URL) of a web page and it stores and organizes all of the retrieved URLs. Web crawler typically acquires data through various applications based on web pages, including web caching and search engines. Traditional tools for web page extraction generate numerous high-quality and efficient solutions, which have been examined extensively. Choudhary et al. [73] have also proposed numerous extraction strategies to address rich Internet applications. 

*(iv) Technology to Capture Zero-Copy (ZC) Packets.* In ZC, nodes do not produce copies that are not produced between internal memories during packet receiving and sending. During sending, direct data packets originate from the user buffer of applications, pass through network interfaces, and then reach an external network. During receiving, the network interfaces send data packets to the user buffer directly. ZC reduces the number of times data is copied, the number of system calls, and CPU load as datagrams are transmitted from network devices to user program space. To directly communicate network datagrams to an address space preallocated by the system kernel, ZC initially utilizes the technology of direct memory access. As a result, the CPU is not utilized. The number of system calls is reduced by accessing the internal memory through a detection program. 

*(v) Mobile Equipment*. The functions of mobile devices have strengthened gradually as their usage rapidly increases. As the features of such devices are complicated and as means of data acquisition are enhanced, various data types are produced. Mobile devices and various technologies may obtain information on geographical location information through positioning systems; collect audio information with microphones; capture videos, pictures, streetscapes, and other multimedia information using cameras; and assemble user gestures and body language information via touch screens and gravity sensors. In terms of service quality and level, mobile Internet has been improved by wireless technologies, which capture, analyze, and store such information. For instance, the iPhone is a “Mobile Spy” that collects wireless data and geographical positioning information without the knowledge of the user. It sends such information back to Apple Inc. for processing; similarly, Google's Android (an operating system for smart phones) and phones running Microsoft Windows also gather such data.

Aside from the aforementioned methods, which utilize technologies and techniques for Big Data, other methods, technologies, techniques, and/or systems of data collection have been developed. In scientific experiments, for instance, many special tools and techniques can acquire experimental data, including magnetic spectrometers and radio telescopes.

### 4.3. Data Analysis

Data analysis enables an organization to handle abundant information that can affect the business. However, data analysis is challenging for various applications because of the complexity of the data that must be analyzed and the scalability of the underlying algorithms that support such processes [74]. Data analysis has two main objectives: to understand the relationships among features and to develop effective methods of data mining that can accurately predict future observations [75]. Various devices currently generate increasing amounts of data. Accordingly, the speed of the access and mining of both structured and unstructured data has increased over time [76]. Thus, techniques that can analyze such large amounts of data are necessary. Available analytical techniques include data mining, visualization, statistical analysis, and machine learning. For instance, data mining can automatically discover useful patterns in a large dataset.

Data mining is widely used in fields such as science, engineering, medicine, and business. With this technique, previously hidden insights have been unearthed from large amounts of data to benefit the business community [2]. Since the establishment of organizations in the modern era, data mining has been applied in data recording. However, Big Data is composed of not only large amounts of data but also data in different formats. Therefore, high processing speed is necessary [77]. For flexible data analysis, Begoli and Horey [78] proposed three principles: first, architecture should support many analysis methods, such as statistical analysis, machine learning, data mining, and visual analysis. Second, different storage mechanisms should be used because all of the data cannot fit in a single type of storage area. Additionally, the data should be processed differently at various stages. Third, data should be accessed efficiently. To analyze Big Data, data mining algorithms that are computer intensive are utilized. Such algorithms demand high-performance processors. Furthermore, the storage and computing requirements of Big Data analysis are effectively met by cloud computing [79].

To leverage Big Data from microblogging, Lee and Chien [80] introduced an advanced data-driven application. They developed the text-stream clustering of news classification online for real-time monitoring according to density-based clustering models, such as Twitter. This method broadly arranges news in real time to locate global information. Steed et al. [81] presented a system of visual analytics called EDEN to analyze current datasets (earth simulation). EDEN is a solid multivariate framework for visual analysis that encourages interactive visual queries. Its special capabilities include the visual filtering and exploratory analysis of data. To investigate Big Data storage and the challenges in constructing data analysis platforms, Lin and Ryaboy [82] established schemes involving PB data scales. These schemes clarify that these challenges stem from the heterogeneity of the components integrated into production workflow.

Fan and Liu [75] examined prominent statistical methods to generate large covariance matrices that determine correlation structure; to conduct large-scale simultaneous tests that select genes and proteins with significantly different expressions, genetic markers for complex diseases, and inverse covariance matrices for network modeling; and to choose high-dimensional variables that identify important molecules. These variables clarify molecule mechanisms in pharmacogenomics.

Big Data analysis can be applied to special types of data. Nonetheless, many traditional techniques for data analysis may still be used to process Big Data. Some representative methods of traditional data analysis, most of which are related to statistics and computer science, are examined in the following sections. 

*(i) Data Mining Algorithms.* In data mining, hidden but potentially valuable information is extracted from large, incomplete, fuzzy, and noisy data. Ten of the most dominant data mining techniques were identified during the IEEE International Conference on Data Mining [83], including SVM, C4.5, Apriori, k-means, Cart, EM, and Naive Bayes. These algorithms are useful for mining research problems in Big Data and cover classification, regression, clustering, association analysis, statistical learning, and link mining. 

*(ii) Cluster Analysis.* Cluster analysis groups objects statistically according to certain rules and features. It differentiates objects with particular features and distributes them into sets accordingly. For example, objects in the same group are highly heterogeneous, whereas those in another group are highly homogeneous. Cluster analysis is an unsupervised research method that does not use training data [3]. 

*(iii) Correlation Analysis.* Correlation analysis determines the law of relations among practical phenomena, including mutual restriction, correlation, and correlative dependence. It then predicts and controls data accordingly. These types of relations can be classified into two categories. (i) Function reflects the strict relation of dependency among phenomena. This relation is called a definitive dependence relationship. (ii) Correlation corresponds to dependent relations that are uncertain or inexact. The numerical value of a variable may be similar to that of another variable. Thus, such numerical values regularly fluctuate given the surrounding mean values. 

*(iv) Statistical Analysis.* Statistical analysis is based on statistical theory, which is a branch of applied mathematics. In statistical theory, uncertainty and randomness are modeled according to probability theory. Through statistical analysis, Big Data analytics can be inferred and described. Inferential statistical analysis can formulate conclusions regarding the data subject and random variations, whereas descriptive statistical analysis can describe and summarize datasets. Generally, statistical analysis is used in the fields of medical care and economics [84]. 

*(v) Regression Analysis.* Regression analysis is a mathematical technique that can reveal correlations between one variable and others. It identifies dependent relationships among randomly hidden variables on the basis of experiments or observation. With regression analysis, the complex and undetermined correlations among variables are simplified and regularized.

In real-time instances of data flow, data that are generated at high speed strongly constrain processing algorithms spatially and temporally; therefore, certain requests must be fulfilled to process such data [85]. With the gradual increase in data amount, new infrastructure must be developed for common functionality in handling and analyzing different types of Big Data generated by services. To facilitate quick and efficient decision-making, large amounts of various data types must be analyzed. The following section describes the common challenges in Big Data analysis.

#### 4.3.1. Heterogeneity

Data mining algorithms locate unknown patterns and homogeneous formats for analysis in structured formats. However, the analysis of unstructured and/or semistructured formats remains complicated. Therefore, data must be carefully structured prior to analysis. In hospitals, for example, each patient may undergo several procedures, which may necessitate many records from different departments. Furthermore, each patient may have varying test results. Some of this information may not be structured for the relational database. Data variety is considered a characteristic of Big Data that follows the increasing number of different data sources, and these unlimited sources have produced much Big Data, both varied and heterogeneous [86].  Table 5 shows the difference between structured and unstructured data.

#### 4.3.2. Scalability

Challenging issues in data analysis include the management and analysis of large amounts of data and the rapid increase in the size of datasets. Such challenges are mitigated by enhancing processor speed. However, data volume increases at a faster rate than computing resources and CPU speeds. For instance, a single node shares many hardware resources, such as processor memory and caches. As a result, Big Data analysis necessitates tremendously time-consuming navigation through a gigantic search space to provide guidelines and obtain feedback from users. Thus, Sebepou and Magoutis [87] proposed a scalable system of data streaming with a persistent storage path. This path influences the performance properties of a scalable streaming system slightly.

#### 4.3.3. Accuracy

Data analysis is typically buoyed by relatively accurate data obtained from structured databases with limited sources. Therefore, such analysis results are accurate. However, analysis is adversely affected by the increase in the amount of and the variety in data sources with data volume [2]. In data stream scenarios, high-speed data strongly constrain processing algorithms spatially and temporally. Hence, stream-specific requirements must be fulfilled to process these data [85].

#### 4.3.4. Complexity

According to Zikopoulos and Eaton [88], Big Data can be categorized into three types, namely, structured, unstructured, and semistructured. Structured data possess similar formats and predefined lengths and are generated by either users or automatic data generators, including computers or sensors, without user interaction. Structured data can be processed using query languages such as SQL. However, various sources generate much unstructured data, including satellite images and social media. These complex data can be difficult to process [88].

In the era of Big Data, unstructured data are represented by either images or videos. Unstructured data are hard to process because they do not follow a certain format. To process such data, Hadoop can be applied because it can process large unstructured data in a short time through clustering [88, 89]. Meanwhile, semistructured data (e.g., XML) do not necessarily follow a predefined length or type.

Hadoop deconstructs, clusters, and then analyzes unstructured and semistructured data using MapReduce. As a result, large amounts of data can be processed efficiently. Businesses can therefore monitor risk, analyze decisions, or provide live feedback, such as postadvertising, based on the web pages viewed by customers [90]. Hadoop thus overcomes the limitation of the normal DBMS, which typically processes only structured data [90]. Data complexity and volume are a Big Data challenge and are induced by the generation of new data (images, video, and text) from novel sources, such as smart phones, tablets, and social media networks [91]. Thus, the extraction of valuable data is a critical issue.

Validating all of the items in Big Data is almost impractical. Hence, new approaches to data qualification and validation must be introduced. Data sources are varied both temporally and spatially according to format and collection method. Individuals may contribute to digital data in different ways, including documents, images, drawings, models, audio/video recordings, user interface designs, and software behavior. These data may or may not contain adequate metadata description (i.e., what, when, where, who, why, and how it was captured, as well as its provenance). Such data is ready for heavy inspection and critical analysis.

### 4.4. Storing/Sharing/Publishing

Data and its resources are collected and analyzed for storing, sharing, and publishing to benefit audiences, the public, tribal governments, academicians, researchers, scientific partners, federal agencies, and other stakeholders (e.g., industries, communities, and the media). Large and extensive Big Data datasets must be stored and managed with reliability, availability, and easy accessibility; storage infrastructures must provide reliable space and a strong access interface that can not only analyze large amounts of data, but also store, manage, and determine data with relational DBMS structures. Storage capacity must be competitive given the sharp increase in data volume; hence, research on data storage is necessary. 

*(i) Storage System for Large Data.* Numerous emerging storage systems meet the demands and requirements of large data and can be categorized as direct attached storage (DAS) and network storage (NS). NS can be further classified into (i) network attached storage (NAS) and (ii) storage area network (SAN). In DAS, various HDDs are directly connected to servers. Each HDD receives a certain amount of input/output (I/O) resource, which is managed by individual applications. Hence, DAS is suitable only for servers that are interconnected on a small scale. Given this low scalability, storage capacity is increased, but expandability and upgradeability are greatly limited.

NAS is a storage device that supports a network. It is connected directly to a network through a switch or hub via TCP/IP protocols. In NAS, data are transferred as files. The I/O burden on a NAS server is significantly lighter than that on a DAS server because the NAS server can indirectly access a storage device through networks. NAS can orient networks, especially scalable and bandwidth-intensive networks. Such networks include high-speed networks of optical-fiber connections. The SAN system of data storage is independent with respect to storage on the local area network (LAN). To maximize data management and sharing, multipath data switching is conducted among internal nodes. The organization systems of data storage (DAS, NAS, and SAN) can be divided into three parts: (i) Disc array, wherein the foundation of a storage system provides the fundamental guarantee; (ii) connection and network subsystems, which connect one or more disc arrays and servers; (iii) storage management software, which oversees data sharing, storage management, and disaster recovery tasks for multiple servers. 

*(ii) Distributed Storage System.* The initial challenge of Big Data is the development of a large-scale distributed system for storage, efficient processing, and analysis. The following factors must be considered in the use of distributed system to store large data.

- *Consistency.* To store data cooperatively, multiple servers require a distributed storage system. Hence, the chances of server failure increase. To ensure the availability of data during server failure, data are typically distributed into various pieces that are stored on multiple servers. As a result of server failures and parallel storage, the generated copies of the data are inconsistent across various areas. According to the principle of consistency, multiple copies of data must be identical in the Big Data environment.
- *Availability.* The distributed storage system operates in multiple sets of servers in various locations. As the numbers of server increase, so does failure probability. However, the entire system must meet user requirements in terms of reading and writing operations. In the distributed system of Big Data, quality of service (QoS) is denoted by availability.
- *Partition Tolerance.* In a distributed system, multiple servers are linked through a network. The distributed storage system should be capable of tolerating problems induced by network failures, and distributed storage should be effective even if the network is partitioned. Thus, network link/node failures or temporary congestion should be anticipated.

### 4.5. Security

This stage of the data life cycle describes the security of data, governance bodies, organizations, and agendas. It also clarifies the roles in data stewardship. Therefore, appropriateness in terms of data type and use must be considered in developing data, systems, tools, policies, and procedures to protect legitimate privacy, confidentiality, and intellectual property. The following section discusses Big Data security further.

#### 4.5.1. Privacy

Organizations in the European Union (EU) are allowed to process individual data even without the permission of the owner based on the legitimate interests of the organizations as weighed against individual rights to privacy. In such situations, individuals have the right to refuse treatment according to compelling grounds of legitimacy (Daniel, 2013). Similarly, the doctrine analyzed by the Federal Trade Commission (FTC) is unjust because it considers organizational benefits.

A major risk in Big Data is data leakage, which threatens privacy. Recent controversies regarding leaked documents reveal the scope of large data collected and analyzed over a wide range by the National Security Agency (NSA), as well as other national security agencies. This situation publicly exposed the problematic balance between privacy and the risk of opportunistic data exploitation [92, 93]. In consideration of privacy, the evolution of ecosystem data may be affected. Moreover, the balance of power held by the government, businesses, and individuals has been disturbed, thus resulting in racial profiling and other forms of inequity, criminalization, and limited freedom [94]. Therefore, properly balancing compensation risks and the maintenance of privacy in data is presently the greatest challenge of public policy [95]. In decision-making regarding major policies, avoiding this process induces progressive legal crises.

Each cohort addresses concerns regarding privacy differently. For example, civil liberties represent the pursuit of absolute power by the government. These liberties blame privacy for pornography and plane accidents. According to Hawks privacy, no advantage is compelling enough to offset the cost of great privacy. However, lovers of data no longer consider the risk of privacy as they search comprehensively for information. Existing studies on privacy [92, 93] explore the risks posed by large-scale data and group them into private, corporate, and governmental concerns; nonetheless, they fail to identify the benefits. Rubinstein [95] proposed many frameworks to clarify the risks of privacy to decision makers and induce action. As a result, commercial enterprises and the government are increasingly influenced by feedback regarding privacy [96].

The privacy perspective on Big Data has been significantly advantageous as per cost-benefit analysis with adequate tools. These benefits have been quantified by privacy experts [97]. However, the social values of the described benefits may be uncertain given the nature of the data. Nonetheless, the mainstream benefits in privacy analysis remain in line with the existing privacy doctrine authorized by the FTC to prohibit unfair trade practices in the United States and to protect the legitimate interests of the responsible party as per the clause in the EU directive on data protection [98]. To concentrate on shoddy trade practice, the FTC has cautiously delineated its Section 5 powers.

#### 4.5.2. Integrity

Data integrity is critical for collaborative analysis, wherein organizations share information with analysts and decision makers. In this activity, data mining approaches are applied to enhance the efficiency of critical decision-making and of the execution of cooperative tasks. Data integrity is a particular challenge for large-scale collaborations, in which data changes frequently. This definition matches with the approach proposed by Clark and Wilson to prevent fraud and error [99]. Integrity is also interpreted according to the quality and reliability of data. Previous literature also examines integrity from the viewpoint of inspection mechanisms in DBMS.

Despite the significance of this problem, the currently available solutions remain very restricted. Integrity generally prevents illegal or unauthorized changes in usage, as per the definition presented by Clark and Wilson regarding the prevention of fraud and error [99]. Integrity is also related to the quality and reliability of data, as well as inspection mechanisms in DBMS. At present, DBMS allows users to express a wide range of conditions that must be met. These conditions are often called integrity constraints. These constraints must result in consistent and accurate data. The many-sided concept of integrity is very difficult to address adequately because different approaches consider various definitions. For example, “Clark and Wilson” addressed the amendment of erroneous data through well-formed transactions and the separation of powers. Furthermore, the Biba integrity model prevents data corruption and limits the flow of information between data objects [100].

With respect to large data in cloud platforms, a major concern in data security is the assessment of data integrity in untrusted servers [101]. Given the large size of outsourced data and the capacity of user-bound resources, verifying the accuracy of data in a cloud environment can be daunting and expensive for users. In addition, data detection techniques are often insufficient with regard to data access because lost or damaged data may not be recovered in time. To address the problem of data integrity evaluation, many programs have been established in different models and security systems, including tag-based, data replication-based, data-dependent, and block-dependent programs. Priyadharshini and Parvathi [101] discussed and compared tag-based and data replication-based verification, data-dependent tag and data-independent tag, and entire data and data block dependent tag.

#### 4.5.3. Availability

In cloud platforms with large data, availability is crucial because of data outsourcing. If the service is not available to the user when required, the QoS is unable to meet service level agreement (SLA). The following threats can induce data unavailability [102].

*(i) Threats to Data Availability.* Denial of service (DoS) is the result of flooding attacks. A huge amount of requests is sent to a particular service to prevent it from working properly. Flooding attacks are categorized into two types, namely, direct DoS and mitigation of DoS attacks [95]. In direct DoS, data are completely lost as a result of the numerous requests. However, tracing first robotics competition (FRC) attacks is easy. In indirect DoS, no specific target is defined but all of the services hosted on a single machine are affected. In cloud, subscribers may still need to pay for service even if data are not available, as defined in the SLA [103].

*(ii) Mitigation of DoS Attacks. *Some strategies may be used to defend against different types of DoS attacks.  Table 6 details these approaches.

#### 4.5.4. Confidentiality

Confidentiality refers to distorted data from theft. Insurance can usually be claimed by encryption technology [104]. If the databases contain Big Data, the encryption can then be classified into table, disk, and data encryption.

Data encryption is conducted to minimize the granularity of encryption, as well as for high security, flexibility, and applicability/relevance. Therefore, it is applicable for existing data. However, this technology is limited by the high number of keys and the complexity of key management. Thus far, satisfactory results have been obtained in this field in terms of two general categories: discussion of the security model and of the encryption and calculation methods and the mechanism of distributed keys.

### 4.6. Retrieve/Reuse/Discover

Data retrieval ensures data quality, value addition, and data preservation by reusing existing data to discover new and valuable information. This area is specifically involved in various subfields, including retrieval, management, authentication, archiving, preservation, and representation. The classical approach to structured data management is divided into two parts: one is a schema to store the dataset and the other is a relational database for data retrieval. After data are published, other researchers must be allowed to authenticate and regenerate the data according to their interests and needs to potentially support current results. The reusability of published data must also be guaranteed within scientific communities. In reusability, determining the semantics of the published data is imperative; traditionally this procedure is performed manually. The European Commission supports Open Access to scientific data from publicly funded projects and suggests introductory mechanisms to link publications and data [105, 106].

## 5. Opportunities, Open Issues, and Challenges

According to McKinsey [8, 48], the effective use of Big Data benefits 180 transform economies and ushers in a new wave of productive growth. Capitalizing on valuable knowledge beyond Big Data is the basic competitive strategy of current enterprises. New competitors must be able to attract employees who possess critical skills in handling Big Data. By harnessing Big Data, businesses gain many advantages, including increased operational efficiency, informed strategic direction, improved customer service, new products, and new customers and markets.

With Big Data, users not only face numerous attractive opportunities but also encounter challenges [107]. Such difficulties lie in data capture, storage, searching, sharing, analysis, and visualization. These challenges must be overcome to maximize Big Data, however, because the amount of information surpasses our harnessing capabilities. For several decades, computer architecture has been CPU-heavy but I/O-poor [108]. This system imbalance limits the exploration of Big Data. CPU performance doubles every 18 months according to Moore's Law [109], and the performance of disk drives doubles at the same rate. However, the rotational speed of the disks has improved only slightly over the last decade. As a result of this imbalance, random I/O speeds have improved moderately, whereas sequential I/O speeds have increased gradually with density.

Information is simultaneously increasing at an exponential rate, but information processing methods are improving relatively slowly. Currently, a limited number of tools are available to completely address the issues in Big Data analysis. The state-of-the-art techniques and technologies in many important Big Data applications (i.e., Hadoop, Hbase, and Cassandra) cannot solve the real problems of storage, searching, sharing, visualization, and real-time analysis ideally. Moreover, Hadoop and MapReduce lack query processing strategies and possess low-level infrastructures with respect to data processing and its management. For large-scale data analysis, SAS, R, and Matlab are unsuitable. Graph lab provides a framework that calculates graph-based algorithms related to machine learning; however, it does not manage data effectively. Therefore, proper tools to adequately exploit Big Data are still lacking.

Challenges in Big Data analysis include data inconsistency and incompleteness, scalability, timeliness, and security [74, 110]. Prior to data analysis, data must be well constructed. However, considering the variety of datasets in Big Data, the efficient representation, access, and analysis of unstructured or semistructured data are still challenging. Understanding the method by which data can be preprocessed is important to improve data quality and the analysis results. Datasets are often very large at several GB or more, and they originate from heterogeneous sources. Hence, current real-world databases are highly susceptible to inconsistent, incomplete, and noisy data. Therefore, numerous data preprocessing techniques, including data cleaning, integration, transformation, and reduction, should be applied to remove noise and correct inconsistencies [111]. Each subprocess faces a different challenge with respect to data-driven applications. Thus, future research must address the remaining issues related to confidentiality. These issues include encrypting large amounts of data, reducing the computation power of encryption algorithms, and applying different encryption algorithms to heterogeneous data.

Privacy is major concern in outsourced data. Recently, some controversies have revealed how some security agencies are using data generated by individuals for their own benefits without permission. Therefore, policies that cover all user privacy concerns should be developed. Furthermore, rule violators should be identified and user data should not be misused or leaked.

Cloud platforms contain large amounts of data. However, the customers cannot physically assess the data because of data outsourcing. Thus, data integrity is jeopardized. The major challenges in integrity are that previously developed hashing schemes are no longer applicable to such large amounts of data. Integrity checking is also difficult because of the lack of support given remote data access and the lack of information regarding internal storage. The following questions must also be answered. How can integrity assessment be conducted realistically? How can large amounts of data be processed under integrity rules and algorithms? How can online integrity be verified without exposing the structure of internal storage?

Big Data has developed such that it cannot be harnessed individually. Big Data is characterized by large systems, profits, and challenges. Thus, additional research is needed to address these issues and improve the efficient display, analysis, and storage of Big Data. To enhance such research, capital investments, human resources, and innovative ideas are the basic requirements.

## 6. Conclusion

This paper presents the fundamental concepts of Big Data. These concepts include the increase in data, the progressive demand for HDDs, and the role of Big Data in the current environment of enterprise and technology. To enhance the efficiency of data management, we have devised a data-life cycle that uses the technologies and terminologies of Big Data. The stages in this life cycle include collection, filtering, analysis, storage, publication, retrieval, and discovery. All these stages (collectively) convert raw data to published data as a significant aspect in the management of scientific data. Organizations often face teething troubles with respect to creating, managing, and manipulating the rapid influx of information in large datasets. Given the increase in data volume, data sources have increased in terms of size and variety. Data are also generated in different formats (unstructured and/or semistructured), which adversely affect data analysis, management, and storage. This variation in data is accompanied by complexity and the development of additional means of data acquisition.

The extraction of valuable data from large influx of information is a critical issue in Big Data. Qualifying and validating all of the items in Big Data are impractical; hence, new approaches must be developed. From a security perspective, the major concerns of Big Data are privacy, integrity, availability, and confidentiality with respect to outsourced data. Large amounts of data are stored in cloud platforms. However, customers cannot physically check the outsourced data. Thus, data integrity is jeopardized. Given the lack of data support caused by remote access and the lack of information regarding internal storage, integrity assessment is difficult. Big Data involves large systems, profits, and challenges. Therefore, additional research is necessary to improve the efficiency of integrity evaluation online, as well as the display, analysis, and storage of Big Data.

## Figures and Tables

**Table 1:** Rapid growth of unstructured data.

Source	Production
YouTube [15]	(i) Users upload 100 hours of new videos per minute (ii) Each month, more than 1 billion unique users access YouTube (iii) Over 6 billion hours of video are watched each month, which corresponds to almost an hour for every person on Earth. This figure is 50% higher than that generated in the previous year

Facebook [16]	(i) Every minute, 34,722 Likes are registered (ii) 100 terabytes (TB) of data are uploaded daily (iii) Currently, the site has 1.4 billion users (iv) The site has been translated into 70 languages

Twitter [17]	(i) The site has over 645 million users (ii) The site generates 175 million tweets per day

Foursquare [18]	(i) This site is used by 45 million people worldwide (ii) This site gets over 5 billion check-ins per day (iii) Every minute, 571 new websites are launched

Google+ [19]	1 billion accounts have been created

Google [20]	The site gets over 2 million search queries per minute Every day, 25 petabytes (PB) are processed

Apple [20]	Approximately 47,000 applications are downloaded per minute

Brands [20]	More than 34,000 Likes are registered per minute

Tumblr [20]	Blog owners publish 27,000 new posts per minute

Instagram [20]	Users share 40 million photos per day

Flickr [20]	Users upload 3,125 new photos per minute

LinkedIn [20]	2.1 million groups have been created

WordPress [20]	Bloggers publish near 350 new blogs per minute

**Table 2:** Hadoop components and their functionalities.

Hadoop component	Functions
(1) HDFS	Storage and replication
(2) MapReduce	Distributed processing and fault tolerance
(3) HBASE	Fast read/write access
(4) HCatalog	Metadata
(5) Pig	Scripting
(6) Hive	SQL
(7) Oozie	Workflow and scheduling
(8) ZooKeeper	Coordination
(9) Kafka	Messaging and data integration
(10) Mahout	Machine learning

**Table 3:** Hadoop usage.

Specified use	Used by
(1) Searching	Yahoo, Amazon, Zvents
(2) Log processing	Facebook, Yahoo, ContexWeb.Joost, Last.fm
(3) Analysis of videos and images	New York Times, Eyelike
(4) Data warehouse	Facebook, AOL
(5) Recommendation systems	Facebook

**Table 4:** MapReduce tasks.

Steps	Tasks
(1) Input	(i) Data are loaded into HDFS in blocks and distributed to data nodes (ii) Blocks are replicated in case of failures (iii) The name node tracks the blocks and data nodes

(2) Job	Submits the job and its details to the Job Tracker

(3) Job initialization	(i) The Job Tracker interacts with the Task Tracker on each data node (ii) All tasks are scheduled

(4) Mapping	(i) The Mapper processes the data blocks (ii) Key value pairs are listed

(5) Sorting	The Mapper sorts the list of key value pairs

(6) Shuffling	(i) The mapped output is transferred to the Reducers (ii) Values are rearranged in a sorted format

(7) Reduction	Reducers merge the list of key value pairs to generate the final result

(8) Result	(i) Values are stored in HDFS (ii) Results are replicated according to the configuration (iii) Clients read the results from the HDFS

**Table 5:** Structured versus unstructured data.

	Structured data	Unstructured data
Format	Row and columns	Binary large objects
Storage	Database Management Systems (DBMS)	Unmanaged documents and unstructured files
Metadata	Syntax	Semantics
Integration tools	Traditional Data Mining (ETL)	Batch processing

**Table 6:** DoS attack approaches.

Defense strategy	Objectives	Pros	Cons
Defense against the new DoS attack [102]	Detects the new type of DoS	(i) Prevents the bandwidth degradation (ii) Ensures availability of service	Unavailability of the service during application migration

FRC attack detection [102]	Detects the FRC attack	No bandwidth wastage	(i) Cannot always identify the attacker (ii) Does not advise the victim on appropriate action

## Acronyms

API: Application programming interface
DAG: Directed acyclic graph
DSMS: Data Stream Management System
GFS: Google File System
HDDs: Hard disk drives
HDFS: Hadoop Distributed File System
IDC: Industrial Development Corporation
NoSQL: Not Only SQL
REST: Representational state transfer
SDI: Scientific Data Infrastructure
SDLM: Scientific Data Lifecycle Management
UDFs: User Defined Functions
URL: Uniform Resource Locator
WSN: Wireless sensor network.
